# Construction Notes

**Published:** 1893-08-12

**Authors:** 


### CONSTRUCTION NOTES.

Claybury Asylum.—The whole of the laundry contract of this asylum was entrusted to Messrs. Thomas Bradford and Co., of London and Manchester, and a very first-class and complete laundry plant has been the result, besides which the comfort and safety of the workers has been well considered and provided for, the working-rooms being well ventilated by means of large exhaust fans, the shafting running underground, and all gearing covered in with wire guards. Bradford "Vowel," twenty washing machines, with steel washing compartments, are used in both the general and foul washhouses, and in the former there are four of these machines mounted on glazed brickwork platforms, with the boiling and rinsing troughs and hydro-extractors on the floor level, the arrangement being similar in principle, but improved in detail, to that fitted up by this firm at the London Hospital in Whitechapel in 1868, besides many other institutions of a similar kind throughout the country. The attendants' washhouse is fitted up with a number of porcelain washing troughs, at which some of the patients will find employment, and two of Bradford's hand-power "Vowel" machines are in this room. The ironing and mangling room is fitted with steam and hand power "Premier" box mangles and a large ironing machine heated by steam, while the receiving and delivery rooms are fitted up with racks, bins, and tables, all of which are excellent specimens of joinery. The engine-house is by the side of the laundry, and here is fixed the fourteen horse-power Bradford engine and a large water heater that utilizes the exhaust steam from the engine and supplies hot water throughout the laundry. We might also mention that there is a separate and complete little laundry for the superintendent and staff.

Aberdeen Royal Infirmary Extensions.—Now that the whole of the wards in the original front portion of the Aberdeen Royal Infirmary have been transferred to the new buildings in the rear, a commencement has been made with the reconstruction of the administrative block. Whilst the skilful architect, Mr. William Kelly, has not caused the simple outline of the building to be altered, yet the changes within doors are considerable, the space thus at hand being admirably taken advantage of. The whole of the present east wing will be converted into an admission department. A commodious waiting-room with two fire-places are at the entrance, on either side of which waiting-room are medical and surgical admission rooms, galleried respectively to accommodate 112 students. Dressing-rooms for males and females are added to these. Connected with this department also is the casualty ward, which is fitted up with sinks and baths, where patients who require it can undergo the preliminary cleansing process. Important improvements will be made in connection with the kitchen arrangements. An entirely new one will be built, the dimensions of which will be 25 feet by 34, and it will have an open roof and cupola. Store-rooms, larders, and milk-houses, all separately planned, lead off from this, as does also the housekeeper's room. The rooms on the ground floor, which occupy the present front of the building facing south, are to be dining-rooms for the staff, of which there will be four, each with a pantry. The upper floors of the building comprise a nurses' home—as completely and admirably planned as is the rest of this extension, each nurse having a cubicle to herself, and there are to be three bath-rooms. Accommodation, but not in connection with this department, will also be provided for the house surgeons, as also for a general sitting-room for their use. A large and airy sitting-room and reading-room for the nurses is also planned. The work is expected to be completed in eight months, the total cost of the reconstruction being estimated at £6,500.

